# Correction of Severe Posterior Tibial Slope in Revision Total Knee Arthroplasty With Cementation and Cone Technology

**DOI:** 10.1016/j.artd.2023.101306

**Published:** 2023-12-30

**Authors:** Christopher G. Salib, Nathan R. Angerett, Zhongming Chen, Jeremy A. Dubin, Ronald E. Delanois

**Affiliations:** LifeBridge Health, Sinai Hospital of Baltimore, Rubin Institute for Advanced Orthopedics, Baltimore, MD, USA

**Keywords:** Cone, Metaphyseal, Tibia, Revision knee, Arthroplasty

## Abstract

Failed primary total knee arthroplasties are becoming more common among a younger, more active patient demographic. Aseptic failures with proximal tibial bone loss, specifically severe posterior tibial collapse, are difficult problems not well described in the literature. There are limited options for reconstructing large defects of the proximal tibia that appropriately restore slope while providing adequate structural support. To our knowledge, this technique to address a large, uncontained posterior proximal tibial defect has not been described in the literature. The purpose of this case report was to detail the surgical technique of how the implementation of cone technology with cementation techniques produced excellent clinical results for a patient with this difficult problem.

## Introduction

Reconstructing bony defects in failed primary total knee arthroplasties (TKAs) remains a challenging problem despite advances in implant design and surgical technique [1,2]. Specifically, addressing the severe posterior tibial slope in aseptic failures is a difficult clinical scenario with varying degrees of collapse. Restoring an adequate slope without over-resection of the proximal tibia is technically challenging and has historically been treated with a number of surgical techniques depending on the size of the defect and other local host factors. The Anderson Orthopaedic Research Institute classification of bone defects in revision TKA is most frequently used, which takes into consideration both the location of the defect and the stability of the implants. This classification can guide treatment and enable preoperative planning on radiographs [3].

Previously reported techniques to address smaller, contained defects included the use of morselized cancellous bone graft [[3], [4], [5]]. Larger segmental defects have been reconstructed with more robust reconstructive options such as impaction grafting [6]. Other techniques for well-contained metaphyseal defects <5 mm have described the use of cementation techniques with or without screw instrumentation due to its availability, familiarity, and versatility in customizing defect size and morphology [1,2,6]. Additionally, other techniques including the use of modular augmentation components or megaprostheses have generally been reserved for treating larger defects that cannot be addressed by salvage techniques alone [7,8]. We present a case here where a patient presented to our clinic with a failed aseptic primary TKA that had severe excessive posterior tibial slope and varus malalignment. We describe a novel technique for restoring the posterior tibial slope using a combination of metaphyseal cone technology and cementation technique. To our knowledge, this technique to address a large uncontained posterior proximal tibial defect has not been described in the literature.

## Patient case

The patient was a 55-year-old female with a body mass index of 31.8 kg/m^2^ and a past medical history of gastric bypass, fibromyalgia, and a smoking history of 5 pack years. The patient previously underwent a right cemented cruciate-retaining TKA with an orthopaedic surgeon at an outside hospital 2 years prior to presentation.

She reported having persistent low-grade pain since the time of her initial surgery; however, it began to gradually increase over the past several months prior to presentation. She began to rely more heavily on assistive devices and sought consultation from our department.

On physical examination, the patient ambulated with an antalgic gait. She had mild varus deformity on her right knee. Her range of motion was 15 degrees shy of terminal extension to 90 degrees of flexion. She did not have any pain with passive range of motion of the knee but was noted to have gross laxity with varus and valgus stress examination. She also had a limb length discrepancy of the affected extremity of 2.5 centimeters using full-length extremity films.

Anteroposterior and lateral radiographs of the right knee showed a cemented cruciate-retaining TKA with gross failure of the tibial component in varus and 50 degrees of extension deformity with evidence of subsidence, significant bony osteolysis, and possible fracture (Fig. 1a-c). Sunrise views were obtained and showed lateral patellar subluxation with no evidence of radiolucencies or lesions. Retrieval of immediate postoperative radiographs of the index procedure were obtained from the patient’s outside primary surgeon for comparison with a finding of a bone defect under the medial aspect of the tibial component that may have contributed to the rapid failure of the implant (Fig. 2a and b). Inflammatory markers, including erythrocyte sedimentation rate and C-reactive protein, and joint aspiration obtained in clinic were negative for infection. After discussion of the risks, benefits, and alternatives of proceeding with revision TKA, the patient agreed to proceed with surgery.

**Figure 1:**
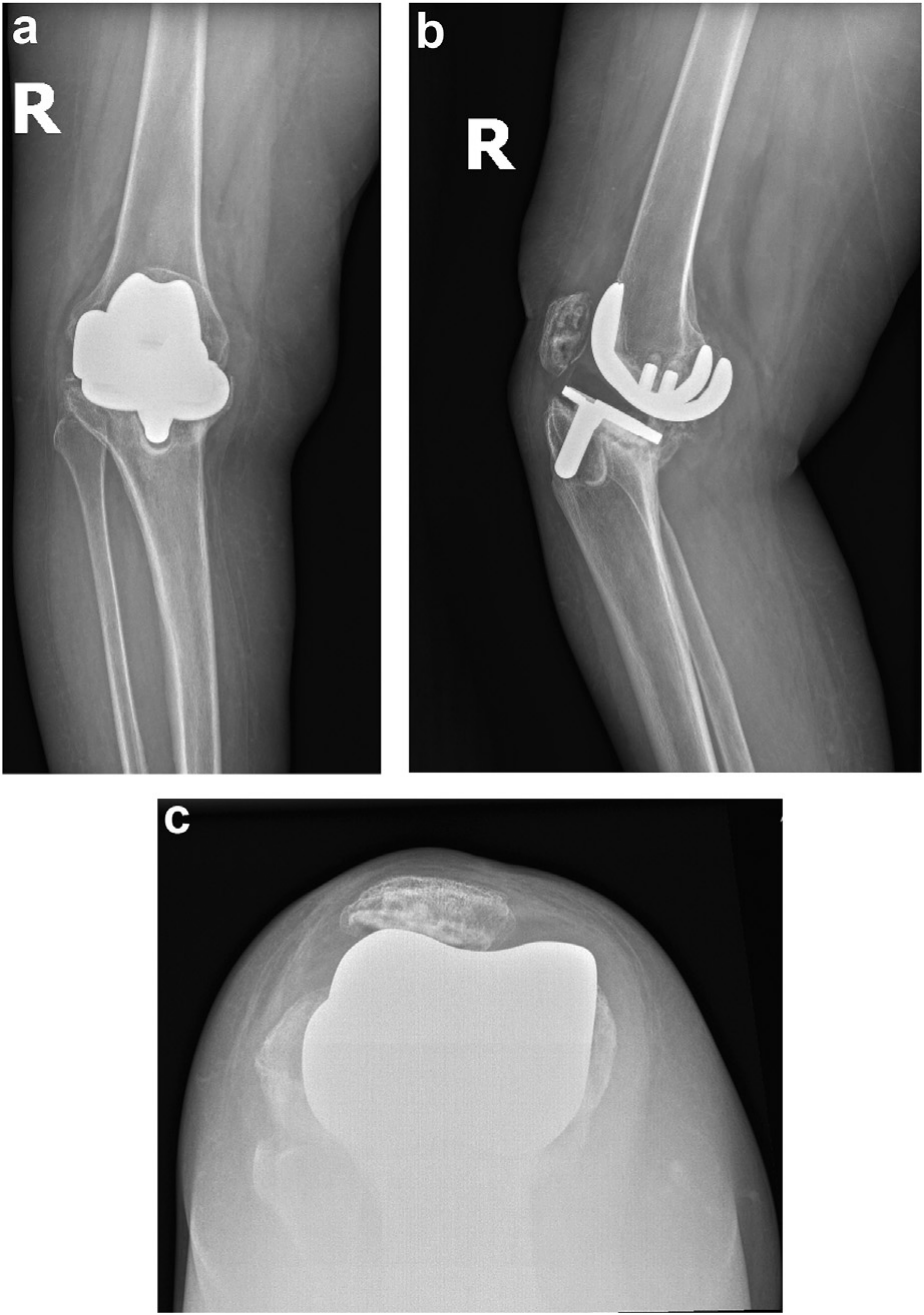
(a) Anteroposterior (AP) radiograph of the right knee demonstrating a failed primary total knee arthroplasty with varus collapse and radiolucency from the tibial keel extending to the medial plateau. (b) Lateral radiograph demonstrating cemented cruciate-retaining TKA with severe posterior collapse of the tibial component into extension. There is radiographic evidence of subsidence, significant bony osteolysis around the tibial component, and possible fracture of the posterior proximal tibia. (c) Sunrise radiographic view demonstrating lateral patellar subluxation.

**Figure 2:**
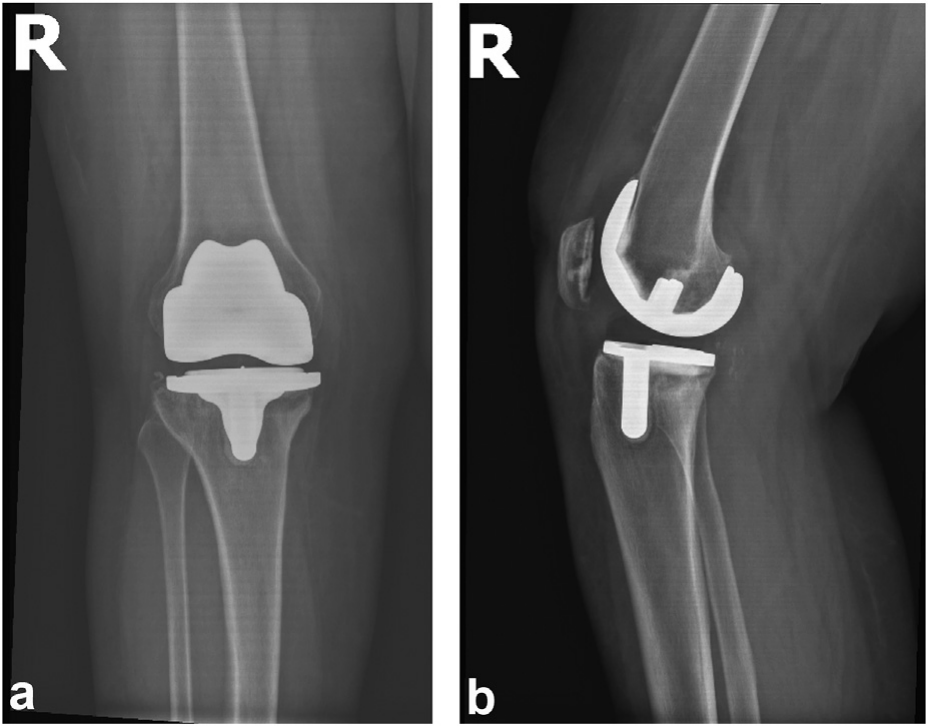
(a) Immediate postoperative AP radiograph following primary TKA obtained from outside facility 2 years prior to presentation in our clinic. (b) Immediate postoperative lateral radiograph following primary TKA demonstrating well-positioned, well-aligned cemented cruciate-retaining TKA system in place.

## Surgical technique

Right knee exposure was achieved by utilizing the patient’s previous midline incision and medial parapatellar approach to enter the joint capsule. A medial and lateral release were performed to allow for femoral exposure, and a Judet quadricepsplasty was performed to address scar adhesions limiting knee flexion beyond 90 degrees.

The femoral component was easily explanted with the femoral component loose prior to the removal attempt by utilizing a small oscillating saw to undermine the anterior flange and then subsequent removal by small bone tamp and mallet while successfully maintaining excellent bony preservation. The tibial baseplate was removed with instruments, and all fibrous tissue was debrided. Intraoperative cultures were sent and came back negative. We evaluated the condition of the tibia, which revealed an approximate 35 mm posterior osseous collapse to the level of the tibial tubercle, with >75% involvement of the tibial plateau. This created a sizable uncontained cavitary defect in the posterior aspect of the proximal tibia.

At this point, we began our reconstruction. We reamed the tibial canal to accept a size 13 stem. A clean-up cut was made in neutral alignment using the intramedullary guide for the entire tibia to restore slope. We then sized and reamed for our cone. Our cone trial was then impacted into place. The cone achieved excellent metaphyseal fixation and served as a stable base for our tibial construct. Additionally, the cone served as a containment wall for our cement in the posterior tibial defect. We then turned our attention to the distal femur. We centrally reamed to a size 16. We needed to open up the flexion gap, so we downsized from a 4 to a 3 femoral component with 4 being the original implant size. We performed our distal femoral resection cut, followed by anterior and posterior chamfer resections, and finally the box resection. Trial femur and tibial components were placed, and intraoperative radiographs confirmed acceptable alignment, implant positioning, and joint height. The knee was well balanced throughout flexion and extension. We removed the trial components, thoroughly irrigated, and inserted the real cone into the proximal tibia utilizing a press fit technique.

Reconstruction options for the tibial defect included more tibial resection, use of bone cement to fill the defect with or without augmentation with screws, bone grafting from autograft or allograft, metal augments, and metaphyseal cones and sleeves. For our patient, metaphyseal cones were utilized.

We then prepared the final tibial and femoral components on the back table. Three bags of Simplex cement (Stryker, Kalamazoo, MI, USA) were used for implant fixation with 1 gram of vancomycin per bag of cement. Prior to cementation, we exsanguinated the leg with an Esmarch and insufflated the tourniquet. Cement was inserted to fill the tibial defect and was gently manipulated in order to restore the appropriate joint line. We inserted the final components into the knee and placed the knee in full extension until the cement was fully cured. The cement mantle was well contained within the tibia. A 9-mm total stabilized polyethylene insert (Stryker, Kalamazoo, MI, USA) was trialed, and the knee was found to be tight medially and loose laterally, so the appropriate medial release was performed. With soft tissue balancing, we achieved balance throughout the patient’s arc of motion, and it was felt to be stable. Excellent patellar tracking was also noted, with an intact extensor mechanism. Standard closure was used to repair the arthrotomy, subcutaneous tissue, and skin.

The patient tolerated the procedure well and was discharged home on postoperative day 2, with weight bearing as tolerated instructions. Her postoperative course was uneventful, and she did not use any assistive devices postoperatively. At her 6-month clinic visit, her pain was completely gone. She was weight-bearing without the use of an assistive device, and her knee range of motion was 0-110 degrees. Her knee was stable to varus and valgus stress examination, and she was very satisfied with her result. She was seen again at her 1-year and 2-year postoperative visits with no complaints and excellently maintained motion and stability of the knee. Radiographs were obtained at her 2-year postoperative visit (Fig. 3a-c).

**Figure 3:**
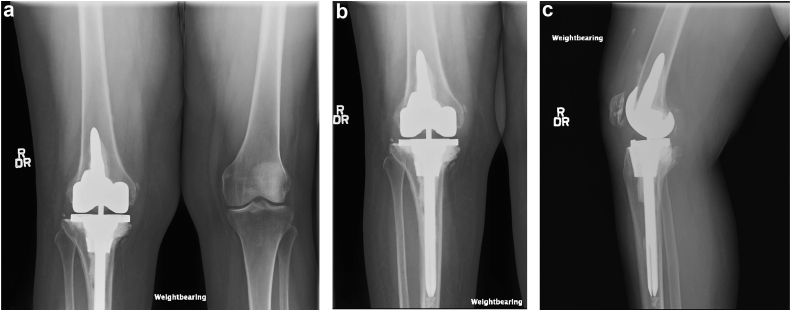
(a) Standing AP radiographs of bilateral knees at the patient’s 2-year postoperative visit show neutral mechanical alignment with well-positioned implants. (b) Standing AP radiographs of the right knee show a well-positioned, well-aligned constrained right TKA with a long-stemmed cemented tibial component and cone augmentation and short-stemmed femoral component. (c) Standing lateral radiograph of the right knee showing maintained alignment of the tibial component in the sagittal plane with cone augmentation and cementation providing adequate structural support to previous proximal tibial defect.

## Discussion

The number of primary TKA failures continues to escalate due to the increasing volume of younger active patients undergoing primary TKA [9,10]. Managing proximal tibia bone loss in aseptic TKA failures remains a challenging scenario for orthopaedic surgeons. Osteolysis surrounding the tibial component may be clinically underestimated by preoperative radiographs, and as such, larger than expected intraoperative bony defects must be anticipated. Scenarios in which the tibia fails due to posterior collapse of the proximal tibial metaphysis are rarely defined explicitly in the literature. In this clinical situation, preserving the joint line is desired, and restoring the height of the posterior tibial metaphysis is the goal without undue bone loss from component removal. Multiple surgical options do exist across the spectrum of severity in this clinical situation and may be employed.

Historically, revision TKAs with larger uncontained defects of the proximal tibia have been managed with allografts, cone augmentations, and endoprostheses. Some studies have reported that even with these allograft reconstructions, they have low rates of osseointegration [8,11]. A series by Bauman et al reviewed 65 patients who underwent revision TKA with bulk allograft for large bony defects and reported 76% survivorship at 10 years. Almost a quarter of patients had failed reconstructions with half due to allograft failure [11]. Similarly, reports on augmented modular components for larger defects are also limited by their high failure rate. Emenari et al published a case report on utilizing stacked porous metaphyseal cones for a severe proximal tibia defect in the revision TKA setting [12]. The authors note that in this particular clinical scenario, it may preclude the use of an offset tibial stem or a stemmed tibial component with a larger diameter than the tibial cone.

Smaller, contained defects have been successfully managed by cementation techniques and are often reinforced with screws for added biomechanical restoration. Many earlier studies have advocated for the use of cement and screws to address tibial defects confined to 5 mm or less [1,2,13]. A large series by Berend et al with a mean follow-up of 7.4 years showed no difference in failure rates between screw and cement fixation compared to no screws in patients undergoing revision of either femoral or tibial components for failed TKA when the osseous defect was >5 mm in size and >50% of plateau or condylar deformity [14]. Advancements in cement technology and surgical technique may be attributed to these results.

The use of cementation in combination with metaphyseal cone augmentation has been described in clinical application in the literature. Indications for this treatment include: massive bone loss comprising a large portion of the femoral condyles or tibial plateaus; and involvement of the collateral ligaments/patellar tendon. Additionally, in cases of excessive femoral bow and severely osteopenic bone, reconstruction is addressed with cement. This approach is avoided in cases where cones can be an irritant to soft tissue, removal is necessary, cement should not be interfaced to implant, and expense is a factor. A cadaveric study by Quevedo Gonzalez et al used finite-element model analysis to identify what combination of metaphyseal cone size and cementation provided the lowest risk of implant-cement debonding and cone-bone micromotion when reconstructing the proximal tibia in the revision TKA setting [15]. They found that despite the reduction in biomechanical stresses at the implant-cement interface in short-stemmed implants, the usage of cones with long, diaphyseal engaging stems were not biomechanically advantageous to short stems. An article by Angerame et al details the surgical technique of utilizing cemented metaphyseal sleeves for revision TKAs and found that for a majority of cases that were categorized as having Anderson Orthopaedic Research Institute 2B bone loss, the all-cause revision-free survivorship was 97.8% at 5.3 years for both tibial and femoral components [16]. Metaphyseal sleeves have the potential advantage of long-term biologic fixation to host bone while creating a stable platform for the implant [16]. Metaphyseal cones may overcome the challenges of sleeves by allowing for independent reestablishment of the joint line, balancing flexion and extension gaps, and restoring metaphyseal defects separately. In the setting of massive femoral and tibial bone loss, management with metaphyseal cones may be advantageous to sleeves, but the decision may be based on surgeon experience, comfort level, as well as geometry of the defect. Our case demonstrates that the use of cementation with a metaphyseal cone and long stem sufficiently reestablished mechanical joint axis alignment in the coronal and sagittal planes while reinforcing adequate structural support of the proximal tibia. While acetabular wedge augments for uncontained tibial defects have been described, a combination of cementation and cone technology to address large uncontained metaphyseal osseous lesions with the need for reestablishing posterior tibial slope has not been previously described [[17], [18]].

There are limitations to this combined cementation and cone technology technique described here. The size of the defect, whether or not morphology of the defect is contained, remaining bone stock quality, and local host factors are all important considerations for employing the proposed technique. Our report notably has short-term follow-up; however, given the excellent clinical outcomes and radiographic results at 2 years, utilizing a metaphyseal cone with cementation may be considered another tool in the surgeon’s armamentarium for these difficult problems.

## Summary

A combination of cementation and cone technology in difficult aseptic revision TKA may be appropriate for certain patients with large, uncontained metaphyseal osseous lesions with the need for reestablishing posterior tibial slope in addition to other anatomic landmarks. This technique has not been described before as a means of restoring the posterior tibial slope.

## Conflicts of interest

R. Delanois receives research support from Biocomposites, CyMedica Orthopedics, Depuy Synthes Product, Flexion Therapeutics, Microport Orthopedics, Orthofix, Patient-Centered Outcomes Research Institute (PCORI), Smith & Nephew, Stryker, Tissue Gene, and United Orthopedic Corporation; all other authors declare no potential conflicts of interest.

For full disclosure statements refer to https://doi.org/10.1016/j.artd.2023.101306.

## Informed patient consent

The author(s) confirm that written informed consent has been obtained from the involved patient(s) or if appropriate from the parent, guardian, power of attorney of the involved patient(s); and, they have given approval for this information to be published in this case report (series).

## CRediT authorship contribution statement

**Christopher G. Salib:** Writing – review & editing, Writing – original draft, Visualization, Data curation. **Nathan R. Angerett:** Writing – review & editing. **Zhongming Chen:** Writing – review & editing. **Jeremy A. Dubin:** Writing – review & editing. **Ronald E. Delanois:** Writing – review & editing, Writing – original draft, Formal analysis, Data curation, Conceptualization.

## Appendix A Supplementary data

Conflict of Interest Statement for Salib

Conflict of Interest Statement for Dubin

Conflict of Interest Statement for Angerett

Conflict of Interest Statement for Delanois

Conflict of Interest Statement for Chen
